# Comment on: “Diabetic retinopathy and diabetic kidney disease, either isolated or associated, impact on the 10-year risk of cardiovascular disease: are we dealing with similar conditions?”

**DOI:** 10.20945/2359-4292-2025-0328

**Published:** 2025-10-08

**Authors:** Karla Dextre-Contreras

**Affiliations:** 1 Instituto de Investigaciones en Ciencias Biomédicas, Universidad Ricardo Palma, Lima, Peru

Dear Editor,

I have read the article entitled “Diabetic retinopathy and diabetic kidney disease, either isolated or associated, impact on the 10-year risk of cardiovascular disease: are we dealing with similar conditions?” and I would like to congratulate the authors, as this research makes a valuable contribution to the growing evidence on diabetes as a relevant risk factor for atherosclerotic cardiovascular disease (ASCVD). However, I would like to offer some comments for further consideration.

The authors have conducted a well-structured cross-sectional study that effectively assesses the 10-year risk of cardiovascular events in patients diagnosed with type 1 or type 2 diabetes mellitus. A significantly higher cardiovascular risk is evident in the groups with kidney involvement and type 2 diabetes mellitus. However, given the limited sample size of patients with type 1 diabetes included in the study, the analysis should be considered an exploration that needs to be expanded in this group of patients ^(1)^.

A strong point of the study was its focus on two specific chronic complications of diabetes (diabetic retinopathy and diabetic kidney disease), which, when present individually, significantly increase the risk of developing ASCVD. It was also found that patients with diabetic kidney disease (with or without diabetic retinopathy) have a higher 10-year risk of ASCVD compared to those without diabetes-related complications. This finding is very important because, according to the International *Diabetes* Federation (IDF) Diabetes Atlas, 90% of patients with diabetes are part of the type 2 diabetes group, and this has led to a silent epidemic of diabetic kidney disease, the leading cause of chronic kidney disease (CKD) worldwide. Even more concerning is its close association with ASCVD, which represents the leading cause of morbidity and mortality in these patients ^(2)^.

The study findings show a high level of accuracy for most of the characteristics analyzed according to the American Heart Association (AHA) Atherosclerotic Cardiovascular Disease Score. However, it would be advisable to document whether the patients had poor glycemic control and interrelated plasma lipid and lipoprotein abnormalities, known as “dyslipidemias”, since, if present, these factors constitute a lethal combination that puts these patients at greater risk for developing cardiovascular disease ^(3)^. These factors, especially cholesterol, would be included in the AHA scoring system, suggesting that assessing them could help improve the system’s accuracy in patients at risk for developing ASCVD. However, the focus of the study on only two specific microvascular complications limits its analysis in this regard. In clinical practice, cholesterol, triglycerids, and glucose control are crucial in determining the risk of diabetes and cardiovascular disease.

Since these factors were not assessed in the study, it is recommended that future research explore the inclusion of both diabetes control and lipid profile optimization, as well as ongoing follow-up to improve the prevention and prognosis of diabetic complications associated with cardiovascular disease. I hope this letter contributes to a broader dialogue about the need to address dyslipidemia in type 2 diabetes as a crucial component in cardiovascular disease prevention.

## Data Availability

datasets related to this article will be available upon request to the corresponding author.
